# Assessment of low-frequency noise from wind turbines under different weather conditions

**DOI:** 10.1007/s40201-020-00478-9

**Published:** 2020-05-21

**Authors:** Chun-Hsiang Chiu, Shih-Chun Candice Lung

**Affiliations:** 1 Research Center for Environmental Changes, Academia Sinica, Taipei, Taiwan; 2 Department of Atmospheric Sciences, National Taiwan University, Taipei, Taiwan; 3 Institute of Environmental Health, National Taiwan University, Taipei, Taiwan

**Keywords:** Wind power, Wind turbines, Low-frequency noise, Noise exposure, Noise model validation

## Abstract

**Background:**

Low-frequency (20–200 Hz) noise (LFN) from wind turbines has received much public attention due to potential health concerns. This work tries to estimate the sound power level of wind turbines (*L*_*W*,A_ (dB)) at 20–200 Hz, which are not provided by manufacturers but essential for estimating LFN exposure (*L*_Aeq_) of nearby residents.

**Methods:**

*L*_*W*,A_ at 20–200 Hz at three wind farms, each with a different brand of wind turbine (Brands A, B and C, respectively) were estimated using propagation model ISO 9613-2 under different weather conditions (rain, wind speed and wind direction) and validated with LFN monitoring data. The feasibility of using validated *L*_*W*,A_ as inputs for ISO 9613-2 to simulate residents’ exposure (*L*_Aeq_) to LFN were assessed considering interferences from rain, wind speed and wind directions.

**Results:**

The average *L*_*W*,A_ at 20–200 Hz ranged between 93.2 and 100.4 dB, 97.8 and 107.2 dB, and 96.5 and 110.4 dB for turbines of Brands A, B, and C, respectively, operating under wind speeds from 2 to 12 m/s. The *L*_*W*,A_ at wind speed of 2–8 m/s increased on average by 1.4, 1.9 and 1.7 dB per 1 m/s increase for Brands A, B and C, respectively. The differences in modeled L_eq_ obtained through the input of *L*_*W*,A_ into the ISO 9613-2 model and the measured *L*_Aeq_ for the three studied wind farms all fall within 1.5 dB.

**Conclusion:**

This study successfully determined and validated the *L*_*W*,A_ of wind turbines of three brands, and subsequent residents’ LFN exposure (with 1.5 dB difference) at three wind farms. Accurately obtaining LFN exposure will serve as the basis for assessing LFN exposure-health relationship. As wind power widely use worldwide, health impact should be assessed based on validated LFN exposure assessment.

**Electronic supplementary material:**

The online version of this article (10.1007/s40201-020-00478-9) contains supplementary material, which is available to authorized users.

## Background

Wind power is used around the world as a source of clean energy. However, wind turbines generate a broad spectrum of low-frequency noise (LFN) in the range of 20–200 Hz [1, 2], which may be audible or inaudible [3–5]. LFN generated from wind turbines can be broadly categorized as aerodynamic noise or mechanical noise [6–8]. With the wind blowing below the cut-in speed, the turbine blades rotate very slowly or are stationary and “parked”, thus generating only minimal noise [7]. Sound power level then increases monotonically when the turbine operates between the cut-in and cut-off wind speed, approximately 4 m/s and 30 m/s, measured at hub height, respectively [7]. The most important component of wind turbine noise emission is aerodynamic noise associated with the passage of air over the blades [1, 6, 7]. Sources of aerodynamic noise include trailing edge noise, impulsive noise, inflow turbulence sound, blade tip noise, and blade-tower interaction [7]. The aerodynamic sound level generally increases with rotor speed, which in turn increases with wind speed.

Studies have widely affirmed that exposure to LFN can have adverse health effects on humans, including annoyance, stress, sleep disturbance, headache, tinnitus, irritation, exhaustion, anxiety, as well as hearing loss, impaired concentration, and in some cases chronic fatigue [1, 9–15]. LFN from wind turbines may cause vibroacoustic disease, characterized by an increased risk of epilepsy, cardiovascular effects, and coronary artery disease [16, 17]. The percentage of people suffering ill effects of LFN increases with increasing noise levels [18–20].

Residents near wind farms are constantly exposed to LFN. Residents’ exposure to turbine LFN can be obtained through either actual measurement or estimation using LFN propagation models. However, a critical parameter of the model, sound power level of wind turbines (*L*_*W*,A_), is neither provided by wind turbine manufacturers, nor found in other documentation. Only *L*_*W*,A_ in the full audio spectrum (audio frequency range of 20–20 kHz) or in certain frequency spectra under 63–8 kHz and 50–10 kHz were provided in some references [21–23]. Since the values of *L*_*W*,A_ at 20–200 Hz cannot be obtained from wind turbine manufacturers, this study estimated this critical parameter, which then served as input for estimating LFN exposure from wind turbines. Knowledge of such can shed light on LFN exposure of residents living near wind farms and contribute to an impact assessment of turbine LFN on residents’ health.

To assess the impact of turbine LFN on residents’ health, their exposure to turbine LFN has to be determined first. Direct measurement of their LFN exposure, though feasible, poses a certain challenge in manpower and resources. In this study, a methodology of estimating LFN exposure of nearby residents was established using a noise propagation model ISO 9613-2. Being a partially empirically based and partially theoretically based noise prediction model, ISO 9613-2 calculates noise levels under conditions favorable to noise propagation [7]. This model is appropriate for modeling propagation of LFN from wind turbines, but the modeling parameters need to be adjusted to account for the unique characteristics of this noise source [24]. The ISO 9613-2 model is commonly used in wind turbine noise calculations [25]. As a standardized and robust noise propagation model [26], ISO 9613-2 is recommended in the Directive 2002/49/EC of the European Parliament and of the Council of the European Union as a national computation method for industrial noise. Not only is ISO 9613-2 modeling fast, it also provides estimations of sound pressure level over a large surface area. In addition, it is integrated into commercially available noise prediction software packages, such as CadnaA [27]. Prior research found consistency between noise levels from wind turbine predicted using CadnaA with analysis results on noise generation data provided by the manufacturer [28]. Contrary to research using commercial software, this study applied directly the model to LFN estimation, thus making the present assessment approach more cost-effective.

However, noise propagation is susceptible to environmental factors and weather conditions, including rain, wind speed, and wind direction [29–32]. According to Ma and Nystuen [33], even light rainfall produces loud sound levels relative to wind. In contrast, Ogunsote suggested that the scattering of sound by rain at ordinary frequencies is insignificant [34]. In view of the inconsistent results on effect of rainfall on noise propagation obtained in prior research, this study attempts to clarify how rainfall affects the propagation of turbine LFN under normal environmental conditions.

Furthermore, multiple LFN sources in the environment can also interfere with noise propagation. LFN is common as background noise in urban environments, generated from many artificial sources: road vehicles, aircraft, rail and sea traffic, industrial machinery, pumps, fans, and air movement machinery such as compressors and ventilation or air-conditioning units [9, 35, 36]. LFN can travel long distances; consequently, the sources can be quite distant.

Taiwan is a small and highly populated island. Wind farms have been set up near residential communities in Taiwan, posing direct impact on daily lives of residents. LFN exposure of the residents’ (*L*_Aeq_) could be estimated based on a propagation model for low frequency which follows ISO 9613-2 principles. However, the required parameter, sound power level of wind turbines (*L*_*W*,A_ (dB)) at 20–200 Hz, of this model was not provided by manufactures. Therefore, this study aims to fill this research gap by estimating the sound power level of turbines (*L*_*W*,A_ (dB)) at 20–200 Hz from the top three companies in the market, which are not provided by manufacturers but essential for estimating LFN exposure of nearby residents. In addition, *L*_*W*,A_ estimates are further validated with field monitoring data.

## Materials and methods

### Wind turbines and wind farms

Wind turbines of three brands, Brands A, B and C, produced by three different manufacturers in the market were selected for this study. These manufacturers account for approximately 30% of the global market share. All brands studied are three-bladed horizontal-axis wind turbines. Table S1 in supplementary material summarizes the environmental setting of the three wind farms studied and the related details of wind turbines operating on the farms.

Wind turbines of Brands A, B and C for three wind farms are located in northern Taiwan. As shown in Fig. S1 in supplementary material, they are NT, TY, and HC located at New Taipei City, Taoyuan and Hsinchu, respectively. Compared with their counterparts in central and southern Taiwan, these three wind farms are located relatively closer to residential areas, which are potential sites of our subsequent health impact assessment of residents under constant exposure to LFN from wind farms in proximity. In addition, the selected wind farms are all located on the western coast of Taiwan where wind is stronger and hence wind speed is higher, compared with the inland areas. While such condition serves best for wind power generation, it would also mean greater noise produced from the turbine.

### Noise propagation model (ISO 9613-2)

The noise propagation model ISO 9613-2 can be employed to predict the propagation of sound power (*L*_*p*_) in ambient sound, construction noise, traffic noise, and wind turbine noise. Of note is that ISO 9613-2 is not valid beyond 1 km [37]; that is, the accuracy of estimate is in question for distances beyond 1 km. This study calculated *L*_*p*_ using the following equation (Eq. 1) and added together the contributions of each 1/3 octave in the range of 20 to 200 Hz [38].

1 $$ {L}_p={L}_{W,A}-20\ \mathrm{dB}\times \log 10\left({\mathrm{d}}_1/1\mathrm{m}\right)-11\mathrm{dB}+{\mathrm{A}}_{\mathrm{gr}}-{\mathrm{A}}_{\mathrm{atm}}\times {\mathrm{d}}_2 $$

where.

*L*_*p*_: 1/3 octave noise at receptor (dB) [In the study, *L*_Aeq_ for 5-min average, *L*_Aeq, 5-min_ dB, is used instead of *L*_*p*_].

*L*_*W*_: 1/3 octave source noise of wind turbine (dB).

d_1_: distance from turbine hub to receptor (m).

A_gr_: frequency-specific ground attenuation (dB).

A_atm_: atmospheric attenuation (dB/km).

d_2_: distance from turbine hub to receptor (km).

When applying the ISO 9613-2 model, A_gr_ can be obtained directly from the frequency-specific ground attenuation table (Table S2 in supplementary material) provided by Saarinen [38], while d_1_ and d_2_ are the measured distances between wind turbines at the wind farm and the fixed monitoring station. A_atm_ is obtained by substituting the temperature and relative humidity measured at the wind farm into Eq. S1 through Eq. S6 in supplementary material [39]. To estimate *L*_*W*,A_, this study measured *L*_*p*_ from wind turbines lying within 1-km radius of the monitoring station. This critical parameter *L*_*p*_ in the ISO 9613-2 model originally stood for peak sound pressure level, but has been replaced with the equivalent continuous sound pressure level *L*_Aeq_ in some studies [24, 40, 41]. As previously indicated, such replacement complies with the simulated noise propagation model. Moreover, it has been reported that at times when the wind turbine sound is dominant, the sound level is relatively constant within 5-min intervals [42]. Hence, the average acoustical intensity over a five-minute period [*L*_Aeq,5min_], measured in A-weighted decibels (dB), is used instead of *L*_*p*_ in Eq. (1).

### LFN monitoring

For continuous measurement of LFN, a hand-held analyzer type NL-62 (Rion, Japan), which complies with IEC 61672–1, 2002 Class 1 and ISO 7196: 1995 applicable standards, was used. The frequency analysis program NX-62RT, with the options of 1/1 octave, 1/3 octave, and broadband sound measurements, was employed to analyze the measurements. In this study, the 1/3 octave noise spectrum with band central frequencies of 20, 25, 31.5, 40, 50, 63, 80, 100, 125, 160, and 200 Hz was used. *L*_Aeq,5min_ were obtained based on these measurements. Measurements were made at intervals of 100 ms and at 1.7 m above the ground, the height close to human ears. To reduce measurement errors due to wind noise at the microphone, especially when making outdoor measurements in windy weather, an all-weather windscreen WS-15 was used. Meteorological data at the three wind farms studied were collected at 2 m above ground using a HOBO weather station (RX3000, Onset Computer Corporation, Bourne, MA), a research-grade, web-enabled and data logging moveable weather station suitable for operation even in harsh outdoor climates. The HOBO weather station comprises a wind speed sensor (S-WSB-M003), temperature/relative humidity sensor (S-THB-M00x), and multi-channel data logger. Five-min meteorological data were analyzed in this study.

Fig. S2 in supplementary material illustrates the locations of wind turbines lying within 1-km radius of the monitoring stations. Only one wind farm was near one monitoring station; the number of wind turbines at each wind farm is listed in Table S1. Only LFN measurements from these turbines were included for estimations as accuracy of ISO 9613-2 estimations is undermined when using measurements obtained at distance beyond 1 km. Although the wind farms are located in different environmental settings, as seen in Table S1, all three monitoring stations are set on the roof of a building surrounded by open space. The height of the building was 5, 6 and 12 m at wind farms NT, TY and HC, respectively. LFN meters and HOBO weather stations were installed at HC in April, at TY in August and at NT in September, 2018. Continuous monitoring was carried out after installation. For comparison, monitoring data of September 2018 were analyzed for most of the data analysis unless specified otherwise.

The starting operational wind speeds of wind turbines are specified as 4 m/s, 3 m/s and 2 m/s for Brands A, B and C, respectively [43–45]. However, it was observed that the wind turbines were turning even below the specified ″operational wind speed″ during the observation period. It was obvious that regular LFN patterns (waves with amplitude of 1–2 dB) occurred due to the running of the wind turbines from examining the noise raw data. In addition, the LFN from wind turbines changed with wind speeds; the observed regular patterns moved gradually up or down according to wind speeds. If certain peaks occurred within these regular patterns, they were presumably from other sources (such as road vehicles and aircraft). Therefore, we removed those sudden peaks from the raw data. Based on these quality control and quality assurance procedures, we are confident that LFN (*L*_Aeq_) in our dataset were from wind turbines rather than from other sources.

### Modeled *L*_Aeq_ compared with measured

This study first estimated *L*_*W*,A_ of turbine LFN input for Eq. (1) to simulate *L*_Aeq_ and then validated the *L*_Aeq_ with actual measurements. To begin with, *L*_Aeq,5min_ was measured at wind farms in September 2018. The measurements served as inputs for the noise propagation model to estimate *L*_*W*,A_ of LFN generated by the wind turbines using Eq. 1. After that, the estimated *L*_*W*,A_ and the measured meteorological parameters (temperature and relative humidity) in October 2018 were input into Eq. 1 to obtain modeled *L*_Aeq,5min_, which was compared with the actual measurements (*L*_Aeq,5min_) in October 2018 to validate the original estimated *L*_*W*,A_ at different wind speeds exceeding 2 m/s.

When inputting the actual *L*_Aeq,5min_ measurements into the ISO 9613-2 model, the difference in height between the turbine hub and the monitoring station was taken into account to calculate the linear distance between the two (d_1_ and d_2_). The difference in height was 45–5 = 40 at NT, 65–6 = 59 m at TY, and 64–12 = 52 m at HC.

In addition, LFN noise from multiple turbines at different distances from the receptor were considered at the same time. Inputting meteorological parameters (temperature and relative humidity) and distance parameters (d_1_ and d_2_) of different turbines (n) into the model produces a system of n equations, the sum of which is the measured *L*_Aeq_. The system of n equations is then solved to obtain the estimated *L*_*W*,A_.

The standard height above open flat ground for measuring wind speed is 10 m [46] and past studies examined variations in *L*_*W*,A_ of wind turbine at wind speeds also at 10 m above ground [28, 47, 48]. In line with the standard and prior practice, this study selected a model developed from wind profile observations in boundary layers which is used in many applications [49–51] to estimate wind speed at 10 m, as required by Eq. (1), from observations made at 2 m above ground.

To investigate the effects of rain on *L*_Aeq_, monitoring data of the studied three wind farms in October to December 2018 were used in addition because there were significantly fewer days of rain in September. The monitoring data (*L*_Aeq_) into the ISO 9613-2 model to estimated *L*_*W*,A_ and that comparing *L*_*W*,A_ between days with and without rain over four months from September through December 2018 was made. From the differences of *L*_*W*,A_ between days with and without rain to clarify effects of rain on *L*_Aeq_. Moreover, the effects of wind speed were examined using monitoring data collected at the three studied wind farms in September 2018, while the effects of different wind directions on *L*_Aeq_ were assessed through comparing data gathered in May (wind direction: southwest, SW) with those in September of 2018 (wind direction: northeast, NE) at HC. Furthermore, the effect of wind directions on noise propagation was evaluated in May and September.

## Results and discussion

Table 1 summarizes the *L*_Aeq,5min_ measured at the wind farms and estimated *L*_*W*,A_ for the three different brands of wind turbines. Taiwan Environmental Protection Administration (EPA) designated different noise standards for different time of the day such as daytime (7 am to 7 pm), evening (7 pm to 10 pm) and nighttime (10 pm to 7 am) [52]. Our observations showed that daytime had higher LFN exposure levels *(L*_Aeq,5min_) at all three wind farms compared to those at evening and nighttime. The *L*_Aeq,5min_ difference between daytime and nighttime were about 1.3–2.9 dB at the three wind farms. Moreover, observed *L*_Aeq,5min_ mainly came from the wind turbines. Nevertheless, there were other possible background noise sources at daytime, resulting in higher *L*_Aeq,5min_. It is obvious that different brands of wind turbines generate different levels of *L*_*W*,A_, with Brand C producing the highest *L*_*W*,A_, implying that the LFN exposure (*L*_Aeq,5min_) in the vicinity of HC would also be the greatest.

**Table 1:** *L*_Aeq, 5-min_ measured at wind farms in September 2018 and estimated *L*_W,A_ for different brands of turbine

Wind farm	Wind Turbine (brand)	*L*_Aeq, 5-min_ measured dB	*L*_*W*,A_ estimated dB	n
(a) 24-hour period				
NT	Brand A	40.6 ± 5.6	89.5 ± 5.8	2473
TY	Brand B	42.7 ± 5.3	95.1 ± 5.4	3521
HC	Brand C	45.2 ± 5.5	96.9 ± 5.4	5012
(b) Daytime (7 am to 19 pm)				
NT	Brand A	41.1 ± 5.2	90.1 ± 5.4	1417
TY	Brand B	43.6 ± 5.3	96.0 ± 5.4	2270
HC	Brand C	46.5 ± 5.1	98.1 ± 4.9	3098
(c) Evening (19 pm to 22 pm)				
NT	Brand A	40.6 ± 5.4	89.5 ± 5.5	270
TY	Brand B	42.8 ± 5.1	95.3 ± 5.1	339
HC	Brand C	44.8 ± 5.6	96.4 ± 5.3	522
(d) Nighttime (22 pm to 7 am)				
NT	Brand A	39.8 ± 6.2	88.7 ± 6.3	786
TY	Brand B	41.4 ± 5.2	93.9 ± 5.2	912
HC	Brand C	43.6 ± 5.6	95.5 ± 5.7	1302

### Effects of rain on *L*_Aeq_ dB

**Fig. 1:**
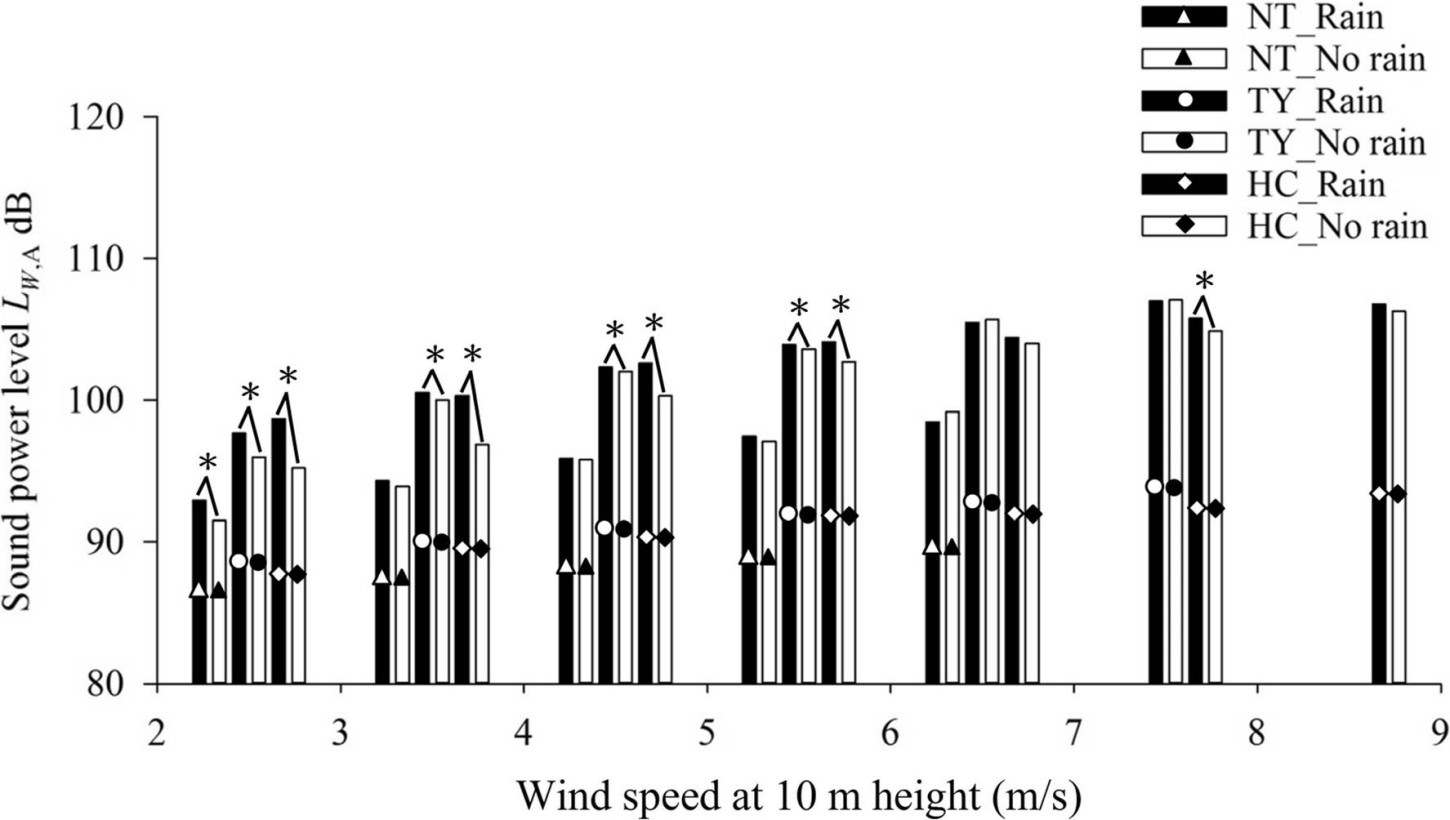
Estimated *L*_*W*,A_ from wind turbines at different wind speeds under “rain” and “no rain” conditions, ^*^Statistically significant at *p* < 0.05 with two-sample t-test

Figure 1 compares the estimated *L*_*W*,A_ from monitoring data (*L*_Aeq_) at different wind speeds between days with and without rain. As can be seen, *L*_*W*,A_ is higher when it rains. Indeed, sound is produced when rain hits against any object, be they buildings, land, water, flowers, trees, including the turbines. Moreover, larger difference in *L*_*W*,A_ of wind turbines is observed at low wind speeds of 2–4 m/s, with the difference becoming smaller at higher wind speeds of 5–9 m/s.

Results of the two-sample t-test, shown in Fig. 1 and Table S3 in supplementary material, indicate significant effect of rain on noise generated in low-wind-speed environments, with the effect decreasing at higher wind speeds. In the case of wind farm NT, the effect of rain on noise generated by Brand A wind turbine was insignificant at wind speed above 3 m/s. Nevertheless, such finding requires further investigation in view of the small number of measurements made under rainy conditions (*n* < 30). Overall, the effect of rain on *L*_Aeq_ at different wind speeds is significant in most cases.

The above analysis implies that beside noise from turbines, wind farm on rainy days will have other sources of environmental LFN. It has been reported that in the presence of rain, rain-generated sound replaces wind-generated noise as the major noise source in the frequency range of 1–50 kHz [33]. In other words, turbine LFN is neither the only nor dominant source of environmental LFN at wind farms under rainy conditions. To rule out the dominant influence of rain on environmental LFN at wind farms and to focus on turbine-generated noise under different wind conditions, only data collected on days without rain were analyzed and are discussed in the following sections.

### Effects of wind speed on *L*_*W*,A_ dB

In northern Taiwan where all three wind farms are located, the prevailing wind comes mainly from northeast (NE) during September to March, and from southwest (SW) during April to August. The measurements for comparison were collected in the same month of September and hence, under the same prevailing NE wind. Only data on days without rain were analyzed.

**Fig. 2:**
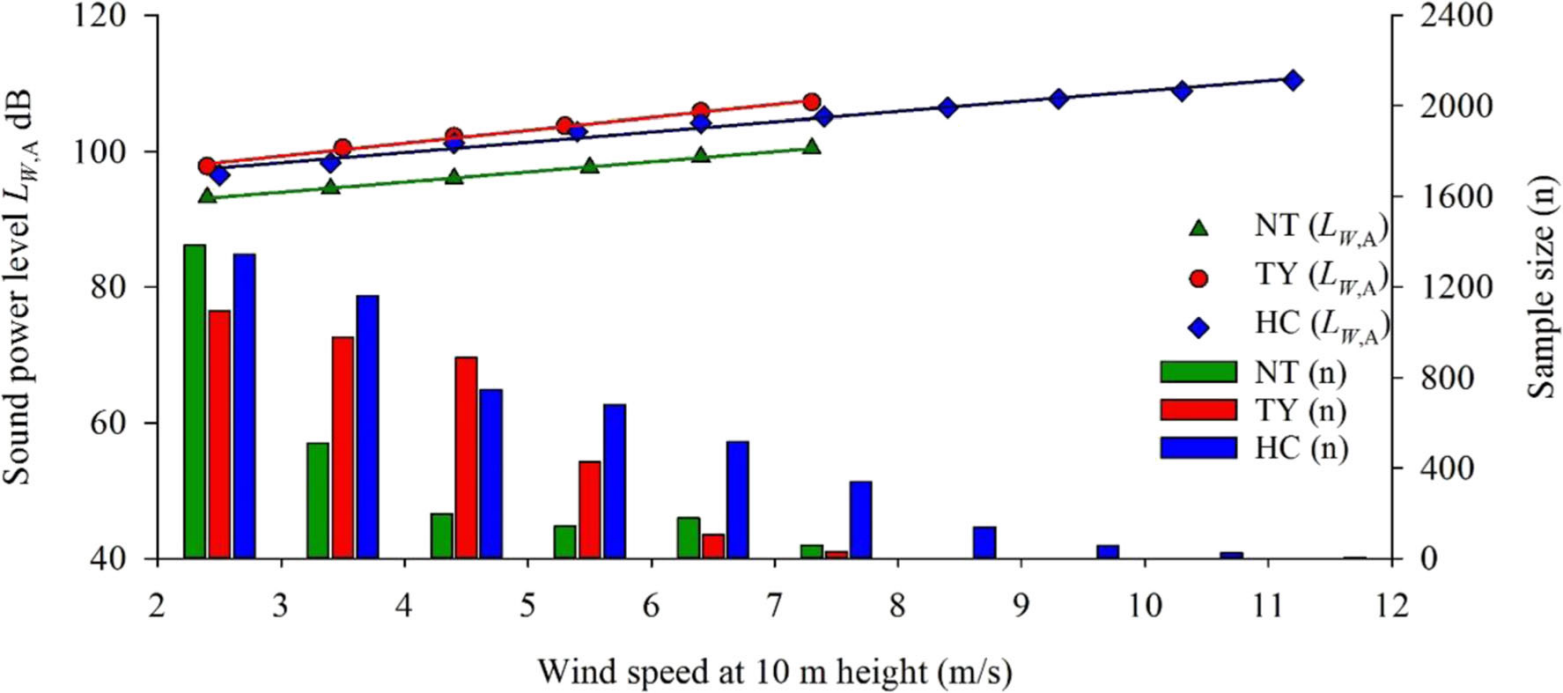
Estimated *L*_*W*,A_ from wind turbines as a function of wind speed at three wind farms in September 2018; the vertical bars are sample sizes with scale shown in the right axis

Figure 2 shows the estimated *L*_*W*,A_ and sample size at wind speeds ranging between 2 and 12 m/s. Sound power levels estimated and number of measurements recorded at different wind speeds are listed in Table S4 in supplementary material. That measurements recorded (*L*_*W*,A_) under three conditions, included 24 h period, Day time and Night time. As shown, the sample size (n) totaled 2473, 3521 and 5012 for NT, TY and HC, respectively. Note that measurements recorded under wind speed of 2 m/s were not included in the analysis. As can be seen, the majority of data collected were within the wind speed range of 2–8 m/s. The total number of measurements collected is in the order of HC > TY > NT, which also implies the frequency of wind speed above 2 m/s recorded during the study period is in the order of HC > TY > NT. Indeed, according to the observation data archived at the Central Weather Bureau, (CWB Observation Date Inquire System, CODiS, https://e-service.cwb.gov.tw/HistoryDataQuery/index.jsp) during September 2018, our study period, the average daily wind speed was 1.1, 2.0 and 2.0 m/s at New Taipei, Taoyuan and Hsinchu area, respectively. Moreover, the highest wind speed recorded was 8 m/s at both NT and TY but 12 m/s at HC. Actual record at CWB of the study period also showed maximum wind speed of 7.4, 8 and 9.2 m/s at New Taipei, Taoyuan and Hsinchu area, respectively. Variations in observations made at the wind farms could be attributed to their geographical locations. NT is more sheltered in the hilly upland in the north; while both TY and HC are along the western shore subject to stronger wind over the sea. Moreover, Hsinchu, where HC wind farm is located, is popularly nicknamed as the ‘Windy City’ for its windy climate with average wind speed over the year 2.4 m/s (CODiS).

Results shown in Fig. 2 reveals good linearity between *L*_*W*,A_ and wind speed; that is, *L*_*W*,A_ increases with increasing wind speeds. In other words, sound power or noise level at turbine is higher at higher wind speed. Although Brand C has the highest *L*_*W*,A_ of 110.4 dB at wind speed of 12 m/s, for the same range of wind speeds, 2–8 m/s, *L*_*W*,A_ generated is in the order of Brand B > Brand C > Brand A. While Brands B and C have similar noise level generated, *L*_*W*,A_ of Brand A is significantly lower, which can be attributed to the smaller wind turbine electric power (≤ 660 kW) of its wind turbine (Table S1 in supplementary material). Moreover, according to the *L*_*W*,A_ displayed in Table S4 in supplementary material, the noise level (*L*_*W*,A_, 24-h period) at wind speed of 2–8 m/s increased on average by 1.4, 1.9 and 1.7 dB per 1 m/s increase for Brands A, B and C, respectively. The daytime *L*_*W*,A_ were 93.3–100.4 dB, 97.8–107.3 dB and 95.7–105.0 dB for Brands A, B and C, respectively, at wind speed of 2–8 m/s. Compared with those at evening and nighttime, the daytime had the highest *L*_*W*,A_ at most wind speed range. In addition, the *L*_*W*,A_ (nighttime) increased on average by 1.4, 1.7 and 1.6 dB per 1 m/s increase for Brands A, B and C, respectively (Table S4). It is mostly consistent with *L*_*W*,A_ increased per 1 m/s at daytime (1.4, 1.9 and 1.8 dB for Brands A, B and C, respectively). Again, the average increase in noise level per 1 m/s increase in wind speed is also in the order of Brand B > Brand C > Brand A, for the same reason of difference in electric power generation. The present findings are consistent with previous results on positive correlation between wind speed and turbine noise emission [30, 31]. Comparison among turbine noise emission at different wind farms under the same wind speed ranges indicate the influence of wind turbine electric power (1500–2300 kW and ≤ 660 kW) on noise generated. This observation also echoes prior reports on more LFN generated by large wind turbines electric power than small ones [1, 53].

Data collected in this study do not allow comparison to be made on noise generation at wind speed exceeding 8 m/s as NT and TY did not record such high wind speed during the study period. Nevertheless, *L*_*W*,A_ at different wind speeds are essential inputs for LFN propagation model for estimating LFN exposures (*L*_Aeq_) of residents under different weather conditions. Furthermore, calculating the R-squared value of *L*_*W*,A_ versus wind speed yielded 0.999, 0.995, and 0.978 for NT, TY and HC, respectively, indicating good correlation between wind turbine noise and wind speed.

### Effects of wind direction on *L*_Aeq_ dB

Among the three wind farms studied, HC had the monitoring station installed in April; hence, measurements obtained there included turbine-generated noise under both NE and SW winds. Moreover, regardless whether the prevailing wind is NE or SW, there are turbines at HC lying upwind and downwind in relation to the monitoring station. As illustrated in Fig. S3 in supplementary material, under SW prevailing wind, two turbines at 795 and 134 m, respectively from the monitoring station sit downwind and one turbine at 322 m from the monitoring station sit upwind of the station; the reverse is true under NE prevailing wind.

**Fig. 3:**
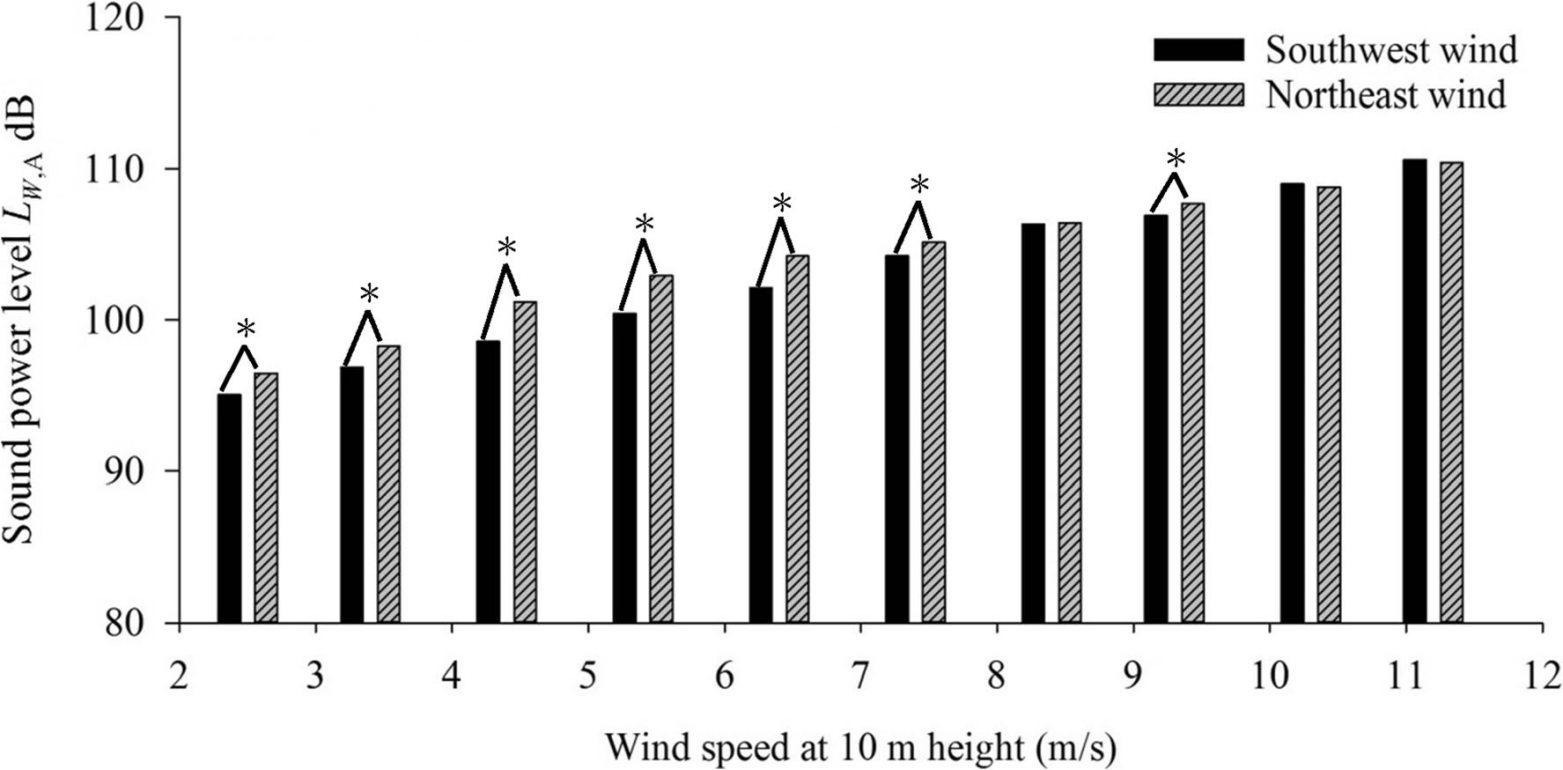
Estimated *L*_*W*,A_ from wind turbines under different wind directions at HC, ^*^Statistically significant at *p* < 0.05 with two-sample t-test

Figure 3 shows the estimated *L*_*W*,A_ of wind turbines under different wind directions at HC. Wind from different directions may affect noise propagation, thus affecting *L*_Aeq_ monitored at our monitoring station. Based on these observed *L*_Aeq_, *L*_*W*,A_ values were estimated based on Eq. (1). As can be seen, estimated *L*_*W*,A_ values are slightly higher under NE wind than under SW wind, with larger difference at wind speeds ranging from 2 to 8 m/s. At higher wind speeds of 8–12 m/s, the difference in estimated *L*_*W*,A_ values at between NE and SW wind narrows, with *L*_*W*,A_ becoming almost identical.

At wind speeds of 2–12 m/s, the difference in estimated *L*_*W*,A_ under the two prevailing winds ranges between 0.1 and 2.6 dB. Evans and Cooper (2012a) reported that the average noise levels measured at 120 m from the turbine did not vary by more than 4 dB (*L*_Aeq_) across all wind directions and the difference in noise propagation between upwind and downwind condition reached 6–7 dB (*L*_Aeq_) at a distance of 1000 m [30]. Measurements made by DeGagne et al. (2002) indicated a difference in turbine noise level of 10 dB (*L*_Aeq_) at a house 500 m away with the wind blowing in opposite directions [54]. In comparison, the difference of 0.1–2.6 dB (*L*_Aeq_) obtained in this study at HC from turbines within 1-km radius is much lower than previous findings. Compare with previous study results that small variation in average noise propagation under different wind directions renders the present approach better suited for simulating LFN exposure (*L*_Aeq_) by the estimated *L*_*W*,A_ from wind turbines of residents in the vicinity of wind farms.

Results of the two-sample t-test, shown in Fig. 3 and Table S5 in supplementary material, revealed significant effect of wind direction on noise propagation in wind speed range of 2–8 m/s. At higher wind speeds of 8–12 m/s, the effect becomes insignificant with the exception of wind speeds of 9–10 m/s. Overall, wind direction had an effect on noise generated at most wind speeds, however, the effect level compare with previous study was insignificant. Moreover, the estimated *L*_*W*,A_ values are higher under prevailing NE than SW wind, which can account for more reports from local residents of louder noise from wind turbines during periods with prevailing NE wind. Therefore, the estimated *L*_*W*,A_ under prevailing NE wind in September of 2018 is served as a basis for future simulations for exposure of residents to LFN from wind turbines.

Difference in measurements obtained between turbine LFN source situated downwind and upwind in relation to the monitoring station can be attributed to the Doppler effect, which indicates an apparent change in sound frequency when a source or receptor of sound is moving relative to the medium. This study focused on monitoring sound within the 20–200 Hz frequency range, and the upwind-downwind difference in turbine LFN sources experienced by the receptors under different wind directions can cause a shift in sound frequency from outside the frequency range into the 20–200 Hz range, resulting in differences in *L*_Aeq_.

### Modeled versus measured *L*_Aeq_ at different wind speeds

**Fig. 4:**
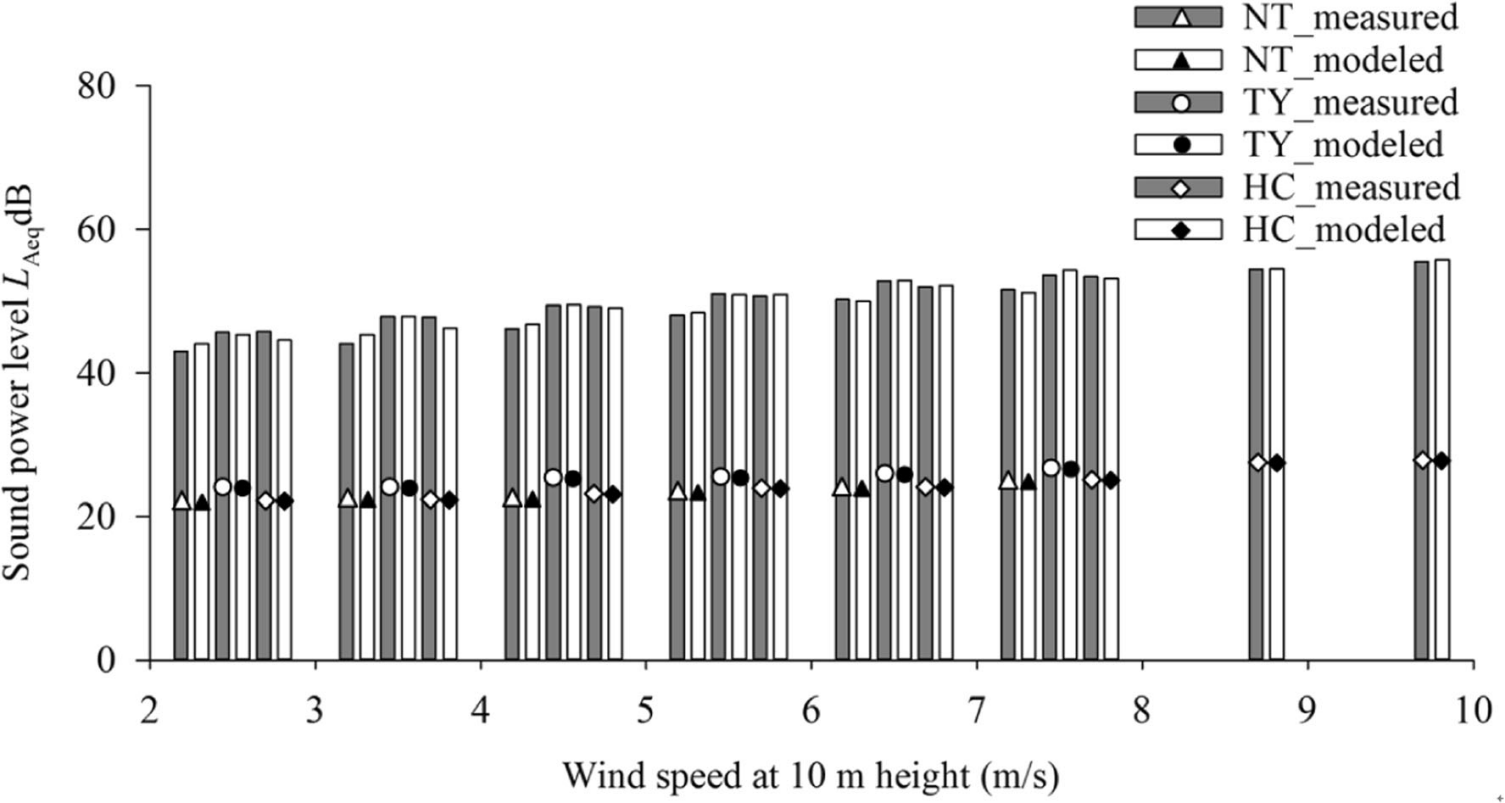
Measured and modeled *L*_Aeq_ (20–200 Hz) in October 2018 from wind turbines at different wind speeds

Figure 4 and Table 2 display the modeled and measured *L*_Aeq_ (20–200 Hz) in October 2018 from wind turbines at different wind speeds. Both modeled and measured results were obtained under similar meteorological conditions including temperature, relative humidity and wind speed. As can be seen, the two results obtained are very close with insignificant differences. The difference between measured and modeled *L*_Aeq_ ranged between 0.6–1.7 dB at NT (2–8 m/s), 0.6–1.6 dB at TY (2–8 m/s), and 0.4–2.4 dB at HC (2–10 m/s). Overall, the difference between modeled *L*_Aeq_ and actual measurement is mostly less than 1.5 dB for LFN propagation at the three wind farms. Our results are consistent with previous studies. Zagubien´ and Ingielewicz (2017) presented consistent results between measurements and computer simulations (by ISO 9613-2 norm). For all wind farms in north and central Poland, the discrepancy between measurements and calculations did not exceed 1.3 dB [55]. In addition, another study showed that noise prediction for wind farms in flat or smooth terrain (such as Carland Cross in England or the Tammhausen in Germany) with a ray-tracing propagation model had good agreement with the measurements [56].

**Table 2:** Difference between measured and modeled *L*_Aeq_ dB (20–200 Hz) of wind turbines at different wind speeds in October 2018

Wind speed (m/s)	NT		TY		HC	
	dB	n	dB	n	dB	n
2–3	1.5 ± 1.3	1238	1.6 ± 1.3	1705	2.0 ± 1.4	2288
3–4	1.7 ± 1.5	216	0.9 ± 0.9	2043	2.4 ± 1.4	1785
4–5	1.2 ± 0.9	117	0.8 ± 0.7	1709	1.7 ± 1.4	1167
5–6	0.9 ± 0.8	84	0.7 ± 0.5	523	1.2 ± 1.1	720
6–7	0.7 ± 0.6	76	0.6 ± 0.4	99	1.3 ± 1.0	305
7–8	0.6 ± 0.5	45	0.8 ± 0.6	5	1.3 ± 0.8	130
8–9					0.6 ± 0.6	14
9–10					0.4 ± 0.5	8

Manual of ISO 9613-2 states a prediction accuracy of ±3 dB for sources of heights up to 30 m above ground and for distances of up to 1000 m from the source [37, 40]. The largest difference between modeled *L*_Aeq_ and actual measurement was 2.4 dB at HC under wind speed 3–4 m/s, which was smaller than ±3 dB stated in the manual. The smaller difference obtained in this study support the validity and accuracy of the present approach in *L*_*W*,A_ estimation. The accuracy of modeled results can be attributed to the environmental setting of the monitoring stations. The absence of buildings or barriers in the open space surrounding the monitoring station allowed uniform propagation of turbine LFN and avoided echo caused by LFN reflecting off barriers.

Residential exposure levels to LFN (*L*_Aeq_) were estimated with *L*_*W*,A_ (dB) as inputs into the ISO 9613-2 model under difference distances. Compare to long distances, receptors at the short distances had higher LFN level (*L*_Aeq_), consistent with a previous study [2]. The distance from the sound source is one of the important factor determining LFN exposure level (*L*_Aeq_) in addition to other surrounding obstacles. It should be also noted that ISO 9613-2 model is valid only within 1 km.

Moreover, the difference between measured and modeled *L*_Aeq_ is larger at low wind speeds of 2–4 m/s but reduces at higher wind speeds exceeding 4 m/s. The presence of other environmental noises in the LFN frequency range accounts for the above differences in results. Smaller *L*_*W*,A_ generated by slow turbine operation at lower wind speeds make these environmental noises seem more apparent, which can affect total LFN. In-depth analysis of the environment around NT and TY reveals their close proximity to roads and the sea. Traffic from both roads (around 260 m and 288 m for NT and TY, respectively) and the sea (around 303 m and 236 m for NT and TY, respectively) are noise sources. Similarly, HC is located close to roads (around 130 m) and the sea (around 162 m) with overhead flight paths. Noises from road, sea and air traffic influence LFN. Previous studies indicated that there are a variety of LFN sources including road vehicles, aircraft, and sea traffic [9, 35, 36]. As wind speed increases, turbine LFN becomes more apparent, and the interference from environmental LFN becomes less noticeable. Turbine LFN at high wind speed drowns out other environmental noises, reducing the difference between measured and modeled *L*_Aeq_. Disturbance to residents comes mainly from higher LFN level at higher wind speed. Accurate estimation of LFN level (*L*_Aeq_) at different distances would serve as a basis for assessing the residents related health impacts.

## Conclusion

This study estimated the *L*_*W*,A_ at 20–200 Hz from wind turbines of three manufacturers (Brands A, B, and C) in order to provide required inputs for ISO 9613-2 to simulate LFN level (*L*_Aeq_) at different distances. Additionally, the effects of weather conditions (rain, wind speed and wind direction) on *L*_Aeq_ were assessed. Our results showed that significant positive effect of rain on noise generated in low-wind-speed environments, with the effect decreasing at higher wind speeds. Average *L*_*W*,A_ at 20–200 Hz ranged between 93.2 and 100.4 dB, 97.8 and 107.2 dB, and 96.5 and 110.4 dB for Brands A, B and C, respectively, at wind speeds of 2–12 m/s. On average, *L*_*W*,A_ at wind speed of 2–8 m/s increased by 1.4, 1.9 and 1.7 dB per 1 m/s increase for Brands A, B and C, respectively. Overall speaking, differences between modeled *L*_Aeq_ and actual measurements are mostly less than 1.5 dB for LFN propagation at the three wind farms. This study successfully determined and validated the *L*_*W*,A_ of wind turbines of three brands, which can serve as a basis for further estimation of residents’ exposure to wind turbine LFN. As wind power widely use worldwide, health impact should be assessed based on validated LFN exposure assessment.

## Electronic supplementary material

ESM 1 (DOCX 6051 kb)
