# Supplementary material for: Assessment of low-frequency noise from wind turbines under different weather conditions
Source: J Environ Health Sci Eng. 2020 May 21;18(2):505–14. doi: 10.1007/s40201-020-00478-9 (PMC7721757; doi:10.1007/s40201-020-00478-9)
Supplement: Supplementary file 1 — (DOCX 6051 kb) [file 40201_2020_478_MOESM1_ESM.docx]

**Supplemental Material**

**Assessment of low-frequency noise from wind turbines under different weather conditions**

Journal of Environmental Health Science and Engineering

Chun-Hsiang Chiu, Shih-Chun Candice Lung^*^

^*^Corresponding author phone: (886) 2-2787-5908. Fax: (886) 2-2783-3584;

E-mail: sclung@rcec.sinica.edu.tw

Address: Research Center for Environmental Changes, Academia Sinica, 128, Section 2, Academia Road, Nankang, Taipei, Taiwan 115.

Number of tables: 5

Number of figures: 3

Number of equations: 6

Table S1 Information of wind farms and wind turbines

| Wind farm | Environment | Wind turbines | | | | | | |
| --- | --- | --- | --- | --- | --- | --- | --- | --- |
|  |  | Brand | Hub  height (m) | Rotor diameter (m) | Number | Power (kW) | Array | Commencement  of operation |
| NT | Hilly upland | A | 45 | 47 | 4 | 660 | Circular | 2004/12 |
| TY | Coastal plain | B | 65 | 70.5 | 20 | 1500 | Linear | 2005/6 |
| HC | Coastal plain | C | 64 | 71 | 5 | 2300 | Linear | 2012/3 |

Table S2 Frequency-specific ground attenuation (Saarinen, 2014)

| Frekvens  (Hz) | Markytans förstärkning  A_gr_ (dB) | Vattenområdets förstärkning A_gr_ (dB) |
| --- | --- | --- |
| 20 | 5.6 | 6.0 |
| 25 | 5.4 | 6.0 |
| 31.5 | 5.2 | 5.9 |
| 40 | 5.0 | 5.9 |
| 50 | 4.7 | 5.8 |
| 63 | 4.3 | 5.7 |
| 80 | 3.7 | 5.5 |
| 100 | 3.0 | 5.2 |
| 125 | 1.8 | 4.7 |
| 160 | 0.0 | 4.0 |
| 200 | 0.0 | 3.0 |

Note: Offshore turbines have a different set of ground attenuations

Table S3 Estimated *L_W_*_,A_ from wind turbines at different wind speeds under “rain” and “no rain” conditions

| Wind speed  (m/s) | NT | | | | TY | | | | HC | | | |
| --- | --- | --- | --- | --- | --- | --- | --- | --- | --- | --- | --- | --- |
|  | Rain | | No rain | | Rain | | No rain | | Rain | | No rain | |
|  | dB | n | dB | n | dB | n | dB | n | dB | n | dB | n |
| 2-3 | 92.9 ± 2.9^a^ | 208 | 91.5 ± 10.0^a^ | 1385 | 97.7 ± 11.1^a^ | 118 | 96.0 ± 12.0^a^ | 1094 | 98.7 ± 4.7^a^ | 51 | 95.2 ± 11.9^a^ | 1345 |
| 3-4 | 94.3 ± 1.4 | 55 | 93.9 ± 3.5 | 510 | 100.5 ± 3.9^a^ | 89 | 100.0 ± 4.7^a^ | 977 | 100.3 ± 4.0^a^ | 55 | 96.9 ± 12.9^a^ | 1161 |
| 4-5 | 95.9 ± 0.9 | 21 | 95.8 ± 1.4 | 198 | 102.3 ± 0.9^a^ | 155 | 102.0 ± 0.9^a^ | 889 | 102.6 ± 2.1^a^ | 58 | 100.3 ± 9.7^a^ | 747 |
| 5-6 | 97.5 ± 0.8 | 9 | 97.1 ± 0.9 | 143 | 103.9 ± 0.6^a^ | 213 | 103.6 ± 0.9^a^ | 427 | 104.1 ± 1.1^a^ | 72 | 102.7 ± 2.6^a^ | 680 |
| 6-7 | 98.5 ± 1.4 | 6 | 99.2 ± 0.5 | 178 | 105.5 ± 0.6 | 67 | 105.7 ± 0.5 | 104 | 104.4 ± 1.9 | 27 | 104.0 ± 1.5 | 515 |
| 7-8 |  |  |  |  | 107.0 ± 0.3 | 6 | 107.1 ± 0.4 | 30 | 105.8 ± 1.3^a^ | 12 | 104.9 ± 1.4^a^ | 339 |
| 8-9 |  |  |  |  |  |  |  |  | 106.8 ± 0.4 | 7 | 106.3 ± 0.9 | 138 |

^a^The differences between “rain” and “no rain” were statistically significant at *p* < 0.05 with two-sample t-test

Table S4 Estimated *L_W_*_,A_ from wind turbines as a function of wind speed at three wind farms in September 2018

| 1. 24-hour period | | | | | | |
| --- | --- | --- | --- | --- | --- | --- |
| Wind speed  (m/s) | NT | | TY | | HC | |
|  | 20-200 Hz | n | 20-200 Hz | n | 20-200 Hz | n |
| 2-3 | 93.2 ± 1.7 | 1385 | 97.8 ± 3.5 | 1094 | 96.5 ± 3.4 | 1345 |
| 3-4 | 94.5 ± 1.2 | 510 | 100.5 ± 2.2 | 977 | 98.3 ± 3.6 | 1161 |
| 4-5 | 96.0 ± 1.2 | 198 | 102.2 ± 0.9 | 889 | 101.2 ± 3.1 | 747 |
| 5-6 | 97.6 ± 0.9 | 143 | 103.7 ± 0.9 | 427 | 102.9 ± 1.6 | 680 |
| 6-7 | 99.2 ± 0.7 | 178 | 105.8 ± 0.7 | 104 | 104.2 ± 1.2 | 515 |
| 7-8 | 100.4 ± 0.6 | 59 | 107.2 ± 0.6 | 30 | 105.1 ± 1.2 | 339 |
| 8-9 |  |  |  |  | 106.4 ± 1.0 | 138 |
| 9-10 |  |  |  |  | 107.7 ± 0.8 | 56 |
| 10-11 |  |  |  |  | 108.8 ± 1.0 | 25 |
| 11-12 |  |  |  |  | 110.4 ± 0.9 | 6 |
| 1. Daytime (7 am to 19 pm) | | | | | | |
| 2-3 | 93.3 ± 1.9 | 868 | 97.8 ± 3.6 | 826 | 95.7 ± 2.8 | 758 |
| 3-4 | 94.5 ± 1.1 | 299 | 100.2 ± 2.5 | 556 | 97.6 ± 3.5 | 816 |
| 4-5 | 95.7 ± 1.3 | 93 | 102.3 ± 1.0 | 496 | 101.0 ± 3.5 | 463 |
| 5-6 | 97.5 ± 0.9 | 59 | 103.6 ± 0.9 | 292 | 103.1 ± 1.4 | 448 |
| 6-7 | 99.1 ± 0.7 | 76 | 105.8 ± 0.8 | 76 | 104.3 ± 1.3 | 313 |
| 7-8 | 100.4 ± 0.5 | 22 | 107.3 ± 0.6 | 24 | 105.0 ± 1.4 | 188 |
| 8-9 |  |  |  |  | 106.4 ± 1.1 | 83 |
| 9-10 |  |  |  |  | 107.7 ± 0.9 | 36 |
| 10-11 |  |  |  |  | 108.8 ± 1.2 | 16 |
| 11-12 |  |  |  |  | 111.0 ± 0.9 | 3 |
| 1. Evening (19 pm to 22 pm) | | | | | | |
| 2-3 | 93.3 ± 1.2 | 164 | 97.8 ± 2.5 | 81 | 97.6 ± 3.7 | 155 |
| 3-4 | 94.3 ± 1.2 | 40 | 100.9 ± 1.4 | 103 | 99.6 ± 2.8 | 102 |
| 4-5 | 96.3 ± 0.8 | 29 | 102.0 ± 1.0 | 90 | 102.0 ± 1.1 | 67 |
| 5-6 | 97.2 ± 1.4 | 3 | 103.8 ± 0.9 | 50 | 102.8 ± 1.0 | 68 |
| 6-7 | 99.3 ± 0.8 | 16 | 106.0 ± 0.7 | 11 | 104.3 ± 0.8 | 77 |
| 7-8 | 100.6 ± 0.7 | 18 | 106.8 ± 0.4 | 4 | 105.3 ± 0.6 | 34 |
| 8-9 |  |  |  |  | 105.9 | 1 |
| 9-10 |  |  |  |  | 107.0 | 1 |
| 10-11 |  |  |  |  |  |  |
| 11-12 |  |  |  |  |  |  |
| 1. Nighttime (22 pm to 7 am) | | | | | | |
| 2-3 | 93.1 ± 1.4 | 353 | 97.8 ± 2.6 | 187 | 97.1 ± 4.1 | 432 |
| 3-4 | 94.5 ± 1.3 | 171 | 100.7 ± 1.5 | 318 | 99.4 ± 3.5 | 243 |
| 4-5 | 96.1 ± 1.1 | 76 | 101.9 ± 0.9 | 303 | 101.4 ± 2.5 | 217 |
| 5-6 | 97.8 ± 0.8 | 81 | 103.9 ± 1.0 | 85 | 102.4 ± 2.0 | 164 |
| 6-7 | 99.3 ± 0.8 | 86 | 105.6 ± 0.4 | 17 | 103.9 ± 1.2 | 125 |
| 7-8 | 100.2 ± 0.5 | 19 | 106.5 ± 0.6 | 2 | 105.0 ± 0.8 | 117 |
| 8-9 |  |  |  |  | 106.4 ± 0.7 | 54 |
| 9-10 |  |  |  |  | 107.7 ± 0.6 | 19 |
| 10-11 |  |  |  |  | 108.8 ± 0.6 | 9 |
| 11-12 |  |  |  |  | 109.7 ± 0.4 | 3 |

Table S5 Estimated *L_W_*_,A_ from wind turbines under different wind directions at HC

| Wind speed  (m/s) | 2018/5  (Southwest wind) | | 2018/9  (Northeast wind) | |  |
| --- | --- | --- | --- | --- | --- |
|  | dB | n | dB | n |  |
| 2-3^a^ | 95.1 ± 7.9 | 1488 | 96.5 ± 11.9 | 1361 |  |
| 3-4^a^ | 96.9 ± 8.5 | 1137 | 98.3 ± 12.9 | 1179 |  |
| 4-5^a^ | 98.6 ± 6.2 | 817 | 101.2 ± 9.6 | 761 |  |
| 5-6^a^ | 100.4 ± 3.3 | 674 | 102.9 ± 2.6 | 687 |  |
| 6-7^a^ | 102.1 ± 4.3 | 377 | 104.2 ± 1.5 | 493 |  |
| 7-8^a^ | 104.2 ± 4.3 | 186 | 105.0 ± 1.4 | 321 |  |
| 8-9 | 106.3 ± 3.1 | 125 | 106.4 ± 1.0 | 138 |  |
| 9-10^a^ | 106.9 ± 2.7 | 84 | 107.7 ± 0.6 | 56 |  |
| 10-11 | 109.0 ± 1.8 | 30 | 108.8 ± 1.0 | 25 |  |
| 11-12 | 110.6 ± 6.0 | 16 | 110.4 ± 0.8 | 6 |  |

^a^The differences between different wind directions were statistically significant at *p* < 0.05 with two-sample t-test

Figure S1 Schematics showing locations of the three wind farms studied

Figure S2 Schematics showing locations of wind turbines within 1-km radius of the monitoring stations at wind farms studied

Figure S3 Schematics showing locations of wind turbines with respect to monitoring station and wind directions at HC

Equations (S1)-(S6). (Source: ISO 9613-1, 1993)

The mechanisms have been extensively studied, empirically quantified, and codified into an

international standard for calculation: ISO 9613-1:1993.

For a standard pressure of one atmosphere, α is absorption coefficient (in dB/m) and can be calculated as a function of frequency *f* (Hz), temperature *T* (degrees Kelvin) and molar concentration of water vapor *h* (%) by Eqs. (S1)~(S3).

$\alpha=8.686\times f^{2}\left\{ 1.84\times{10}^{-11}\left( \frac{T}{T_{0}} \right)^{1/2}+\left( \frac{T}{T_{0}} \right)^{-5/2}\left[ 0.01275\frac{e^{-2239.1/T}}{F_{r,O}+f^{2}/F_{r,O}}+0.1068\frac{e^{-3352/T}}{F_{r,N}+f^{2}/F_{r,N}} \right] \right\}$ (S1)

$F_{r,O}=24+4.04\times{10}^{4}h\frac{0.02+h}{0.391+h}$ (S2)

$F_{r,N}=\left( \frac{T}{T_{0}} \right)^{-1/2}\left( 9+{280he}^{\left\{ -4.17\left[ \left( \frac{T}{T_{0}} \right)^{-1/3}-1 \right] \right\}} \right)$ (S3)

where

F_r,o_: Oxygen relaxation frequency (Hz)

F_r,N_: Nitrogen relaxation frequency (Hz)

T_0_: 293.15^o^K (20^o^C)

In Eqs. (S2) and (S3) for F_rO_ and F_rN_, **h** is equivalent to the molar concentration of water vapor, as a percentage, and is computed as follows:

$\mathbf{h}=h_{\mathrm{rel}}\left( p_{\mathrm{sat}}/p_{r} \right)\left( p_{a}/p_{r} \right)^{-1}$ (S4)

**h_rel_** = relative humidity in percent (either test- or reference-day relative humidity, as appropriate)

$\mathbf{p}_{\mathbf{sat}}=\left( p_{r} \right){10}^{v}$ (S5)

**p_a_** = ambient atmospheric pressure in kPa (either test- or reference-day pressure, as appropriate)

**p_r_** =101.325 kPa, reference pressure of one standard atmosphere

$\mathbf{V}=10.79586\left[ 1-\left( T_{01}/T \right) \right]-5.02808\times\log_{10} \left( T/T_{01} \right)+1.50474\times{10}^{-4}\left\{ 1-{10}^{-8.29692\left[ \left( T/T_{01} \right)-1 \right]} \right\}+0.42873\times{10}^{-3}\left\{ -1+{10}^{4.76955\left[ 1-\left( T_{01}/T \right) \right]} \right\}-2.2195983$ (S6)

**T_01_**=273.16 °K, triple-point isotherm temperature
